# Time‐calibrated relationships of a rare cave catfish ( *Trichomycterus rubbioli* ): Shedding light on troglobitic lifestyle origin in the Brazilian caatinga

**DOI:** 10.1111/jfb.70419

**Published:** 2026-03-25

**Authors:** Wilson J. E. M. Costa, Paulo J. Vilardo, José Leonardo O. Mattos, Axel M. Katz, Valter M. Azevedo‐Santos, Pedro L. C. Uzeda, Rodrigo L. Ferreira

**Affiliations:** ^1^ Laboratory of Systematics and Evolution of Teleost Fishes Institute of Biology, Federal University of Rio de Janeiro Rio de Janeiro Brazil; ^2^ Programa de Pós‐graduação em Biodiversidade, Ecologia, e Conservação Universidade Federal do Tocantins Porto Nacional Brazil; ^3^ Laboratório de Ecologia de Peixes, Departamento de Ecologia e Conservação Universidade Federal de Lavras Lavras Brazil; ^4^ Departamento de Ecologia e Conservação, Centro de Estudos em Biologia Subterrânea, Instituto de Ciências Naturais Universidade Federal de Lavras Lavras Brazil

**Keywords:** cave biodiversity, Cenozoic climate change, Rio São Francisco basin, Serra do Ramalho

## Abstract

Catfishes of the subfamily Trichomycterinae comprise the most diverse fish group with species adapted to live in Neotropical caves, but past evolutionary scenarios that have driven the origin of these troglobitic species remain unknown. We herein investigate the phylogenetic position of the cave‐restricted *Trichomycterus rubbioli*, endemic to the semi‐arid Caatinga of northeastern Brazil, through a time‐calibrated molecular phylogenetic analysis of a broad sample of trichomycterine taxa (84 spp), using two nuclear and two mitochondrial genes (3030 bp). The analysis supported *T. rubbioli* as a member of the *Trichomycterus s.s.* clade endemic to eastern South America and an early Miocene origin for the *T. rubbioli* lineage. The combination of available data suggests that the *T. rubbioli* lineage adapted to the troglobitic lifestyle between the Middle and Late Miocene, a period when the semi‐arid conditions of the Caatinga biome became established and consequently streams of the region became seasonal. This past scenario would be responsible for extinction of epigean trichomycterines while preserving the lineage within the cave system. Morphological characters of the troglobitic *‘Trichomycterus’ itacarambiensis* from the transition area between the Cerrado and the Caatinga indicate distant relationships with *T. rubbioli*, suggesting an independent evolutionary event of cave colonization.

## 1 INTRODUCTION

The wide range of tropical teleost fish adaptations to live in a great variety of environments has attracted the attention of biologists since the last century (e.g. Goulding, 1980; Lowe‐McConnell, 1987). The Neotropical Region ranks among the world's most diverse areas for freshwater teleosts, boasting an extensive hydrographic network that supports species adapted to a wide range of habitats (Albert & Reis, 2011), including subterranean streams (e.g. Trajano, 2021). The fish group with the largest number of species adapted to live in South American caves is Trichomycterinae (Bichuette & Gallão, 2021), a catfish subfamily typically found in fast‐flowing streams (Costa, 2021). Records of troglobitic trichomycterines are scattered across much of South America, with a higher species concentration in the Andes (e.g. Castellanos‐Morales, 2007, 2008, 2010, 2018; DoNascimiento & Prada‐Pedreros, 2020; Mesa et al., 2018). In spite of the large number of records, we still do not know evolutionary past scenarios that may have favoured the origin of troglobitic trichomycterines.

In eastern South America, the cave‐restricted trichomycterine *Trichomycterus rubbioli* Bichuette & Rizzato, 2012 from the Rio São Francisco basin, northeastern Brazil (Bichuette & Rizzato, 2012), has unusual morphological and ecological characteristics among congeners from eastern South America. This species was described prior to phylogenetic studies on trichomycterines, during a period when the geographically widespread genus *Trichomycterus* Valenciennes 1832 which ranged from southern Central America to southern South America was still regarded as a paraphyletic group (Katz et al., 2018; Ochoa et al., 2017, 2020). Subsequently, *Trichomycterus* was restricted to a monophyletic group endemic to eastern South America, encompassing the type species of the genus *Trichomycterus nigricans* Valenciennes, 1832 (Katz et al., 2018). Today, *Trichomycterus* (sensu Katz et al., 2018, hereafter *Trichomycterus s.s*.) comprises more than 80 species distributed between northeastern and southern Brazil (e.g. Vilardo et al., 2023). Since *Trichomycterus s.s*. is not diagnosable by unique morphological characteristics and *T. rubbioli* has not been included in any phylogenetic analysis to date, its position among trichomycterines is still uncertain.

*Trichomycterus rubbioli* occurs in a cave system in the Serra do Ramalho, situated in a karstic area about 500–600 m asl surrounded by ephemeral streams and perennial rivers draining the left bank of the Middle Rio São Francisco, considered the richest hotspot of subterranean biodiversity in the tropics (Ferreira et al., 2023; Vaz et al., 2025). Therefore, perennial fast‐flowing streams, which constitute the typical habitat of trichomycterines, are absent from the surrounding areas. Moreover, the area of occurrence of *T. rubbioli* lies over 250 km away from the geographically nearest records of other *Trichomycterus* species. Furthermore, *T. rubbioli* has morphological features not observed in its congeners. In addition to possessing rudimentary eyes and lacking skin pigmentation, traits that have arisen convergently in different lineages of cave‐restricted fishes, *T. rubbioli* has an adipose fin, the pelvic‐fin base is posterior to the dorsal‐fin origin and the anal‐fin origin is situated at a vertical much posterior to the dorsal‐fin base. In other species of *Trichomycterus s.s*. there is no adipose fin, the pelvic‐fin base is never posterior to the dorsal‐fin origin and the anal‐fin origin is situated at a vertical through the dorsal‐fin base or immediately posterior to it (Costa, 2021).

The distinctive features exhibited by *T. rubbioli* raise doubts as to whether it is in fact a species belonging to the *Trichomycterus s.s*. clade or instead represents a lineage within another trichomycterine group. Here we conducted a multilocus phylogenetic analysis including *T. rubbioli* in a broad sample of *Trichomycterus s.s*. and other trichomycterine species to infer its phylogenetic position. Additionally, we performed a time‐calibrated phylogenetic analysis to infer the time of origin of the *T. rubbioli* lineage.

## 2 MATERIALS AND METHODS

### 2.1 Ethics statement

Field collections were made with permits given by the Instituto Chico Mendes de Conservação da Biodiversidade (ICMBio; permit number: 94534). Field methods were approved by the CEUA‐CCS‐UFRJ (Ethics Committee for Animal Use of Federal University of Rio de Janeiro (CEUA‐CCS‐UFRJ; permit number: 084/23).

### 2.2 Specimens

Specimens of *T. rubbioli* were collected using small dip nets used in aquarium keeping. All specimens were collected in Brazil: the Estado da Bahia, Município de Carinhanha, Serra do Ramalho, Rio São Francisco basin. Specimens used in morphological identification were first fixed in formalin for 2 weeks and then placed in 70% ethanol. To examine bone morphology, one specimen (UFRJ 14299) was cleared and stained using Taylor and Van Dyke (1985). The specimen used in the molecular analysis (UFRJ 14097) and another specimen (UFRJ 14221) were fixed and preserved in absolute ethanol. Specimens were deposited in the ichthyological collection of the Institute of Biology, Federal University of Rio de Janeiro: UFRJ14097, 1 ex.; UFRJ 14247, 3 ex.; UFRJ 14299, 1 ex.; Gruna do Pedro Cassiano, 13°47′45″ S 48°54′46″ W; UFRJ 14221, 1 ex.; Lapa do Peixe, 13°48′03″ S 48°57′05″ W.

### 2.3 DNA extraction, amplification and sequencing

The genomic DNA was extracted from the muscle tissue removed from the right side of the caudal peduncle using a DNeasy Blood & Tissue Kit (Qiagen) following the standard protocol. The DNA extraction product was evaluated using 1% agarose gel electrophoresis. Polymerase chain reaction (PCR) was performed to amplify target DNA gene fragments using the following primers: CYTB Siluri F and CYTB Siluri R (Villa‐Verde et al., 2012) for mitochondrially encoded cytochrome b (CYTB); FISH F1 and FISH R1 (Ward et al., 2005) for mitochondrially encoded cytochrome c oxidase I (COX1); RAG2 TRICHO F and RAG2 TRICHO R (Costa et al., 2020) for recombination activating 2 (RAG2) and MYH6 TRICHO F and MYH6 TRICHO R (Costa et al., 2020) for myosin heavy chain 6 (MYH6). PCR were performed in 60 μL reactions with the following reagent concentrations: 5× Green GoTaq G2 Hot Start Reaction Buffer (Promega), 2.5 mM MgCl2, 1 μM of each primer, 75 ng of total genomic DNA, 0.2 mM of each dNTP and 1 U of GoTaq G2 Hot Start polymerase (Promega). The thermocycling profiles were as follows: initial denaturation at 95°C for 4 min; 35 cycles of denaturation at 94°C for 1 min, annealing at 45–60°C for 1 min and extension at 72°C for 1–1.5 min. Negative controls were included to check for DNA contamination. The PCR products were purified using the Wizard SV Gel and PCR Clean‐Up System (Promega) following the standard protocol. Sanger sequencing reactions were made by Instituto SENAI de Inovação em Biossintéticos. Sequencing chromatograms were assessed using MEGA 12 (Kumar et al., 2024) and the annotated sequences were generated by assembled readings. All generated sequences were translated into amino acids to verify the absence of premature stop codons or indels.

### 2.4 Phylogenetic analyses

DNA sequences for the phylogenetic analyses comprised new sequences of *T. rubbioli* and sequences already available in GenBank. The list of specimens and their respective GenBank accession numbers is provided in Appendix S1. Terminal ingroup taxa used in the analyses included *T. rubbioli*, 62 species of *Trichomycterus s.s*. and 21 trichomycterines representing all other main lineages along their South American distribution. Terminal outgroups comprised eight taxa belonging to other Trichomycteridae genera and six representatives of other Ostariophysian lineages. The analyses were rooted in the characiform *Brevidens striatus* (Kner, 1858). See Vilardo et al. (2023) for justification for outgroup selection. Alignment was conducted in Clustal W (Chenna et al., 2003) algorithm implemented in UniPro UGENE (Okonechnikov et al., 2012). The concatenated dataset was 3030 pb (1088 for CYTB; 521 for COI; 543 for MYH6; 878 for RAG2). The dataset was analysed using a maximum likelihood (ML) approach in IQ‐TREE 2 (Minh et al., 2020), with partitions including each codon position for each gene, for which the best‐fitting models of molecular evolution were calculated using the Bayesian information criterion (BIC) of ModelFinder (Kalyaanamoorthy et al., 2017), implemented in IQ‐TREE 2. The list of partitions and their respective models of nucleotide substitution are presented in the Appendix S2. The method for assessing the reliability of internal branches used in the ML analysis was the ultrafast bootstrap support (UFBoot) (Hoang et al., 2018; Minh et al., 2013), using 1000 replicates and default parameters as implemented in IQ‐TREE 2. The concatenated dataset was additionally analysed using Bayesian inference (BI) with MrBayes 3.2.5 (Ronquist et al., 2012) implemented in CIPRES (Miller et al., 2010), using the best partition scheme and best‐fit models of substitution identified according to the BIC of IQTREE2, with the following parameters: two independent Markov chain Monte Carlo (MCMC) runs of two chains each for 40 million generations, with a tree sampling frequency of every 1000 generations. The convergence of MCMC chains and the burn‐in value were assessed by evaluating the stationary phase of the chains using Tracer 1.7.1 (Rambaut et al., 2018). The BI final consensus tree and the Bayesian posterior probabilities were generated with the remaining tree samples after removing the first 25% of the samples as burn‐in.

### 2.5 Divergence time‐estimation

The same dataset as above was also used to implement the divergence time estimation. The analysis was performed in BEAST v.1.10.4 (Suchard et al., 2018) using the uncorrelated relaxed molecular clock, a Yule speciation process for the tree prior (Gernhard, 2008) and all clade‐age inferences are presented as 95% highest posterior density (HPD). We included four calibration points to constrain divergence dates in our phylogenetic tree. The first point of calibration followed Roxo et al. (2019) based on fossil evidence and previous phylogenetic analyses (Lundberg, 1993; Lundberg et al., 2007; Sullivan et al., 2006) supporting an origin for Siluriformes during the Lower Cretaceous period (145–100 Mya), thus implementing as a lognormal distributed prior, with an offset of 140 million years ago (Mya) and a standard deviation of 14 million years, with this date estimate implemented in the root of the phylogeny. A second calibration point was implemented in the origin of Trichomycteridae, about 106 million years ago (standard deviation = 7) as estimated by Betancur‐R et al. (2015). A third calibration point was the fossil of *Hoplisoma revelatum* (Cockerell, 1925), the oldest known loricarioid fossil (Cockerell, 1925). As in previous studies (Cardoso et al., 2019; Roxo et al., 2014, 2019; Silva et al., 2016; Vilardo et al., 2023), we implemented a log normal prior to 55 Mya (standard deviation = 1) to establish the origin of the Corydoradinae lineage (node including *Corydoras stenocephalus* Eigenmann & Allen, 1942), *Hoplisoma panda* (Nijssen & Isbrucker, 1971) and *Osteogaster aeneus* (Gill, 1858) (Roxo et al., 2014; Vilardo et al., 2023). Finally, the last calibration point was implemented at the divergence between the monotypic subgenus *Plesioscleronema* (Costa et al., 2022) and the subgenus *Scleronema* Eigenmann, 1917, using a calibration age of 4.5 MA (standard deviation = 2.0), corresponding to the minimum age of a trichomycterine fossil identified as a member of the latter subgenus (Costa et al., 2022) from the middle Miocene of Monte Hermoso Formation in Argentina (Bogan & Agnolin, 2009). Tomassini et al. (2013) estimated the upper and lower boundaries of the Monte Hermoso Formation to be 4.5–5.3 MA. To establish the age of this node, we adopted the same parameters used by Ochoa et al. (2017). Two independent runs of MCMC, each run with 60 million of generations were performed with sample frequency of 1000. The value of parameters of the analyses, convergence of the MCMC chains, effective sample size and the stationary distribution were evaluated using Tracer v. 1.7.1. Generated trees were combined in LogCombiner v.1.10.4 (Suchard et al., 2018) after applying a burn‐in of the first 25% in each run. TreeAnnotator v.1.10.4 (Suchard et al., 2018) was used to obtain the maximum credibility tree and posterior probabilities.

## 3 RESULTS

**FIGURE 1:**
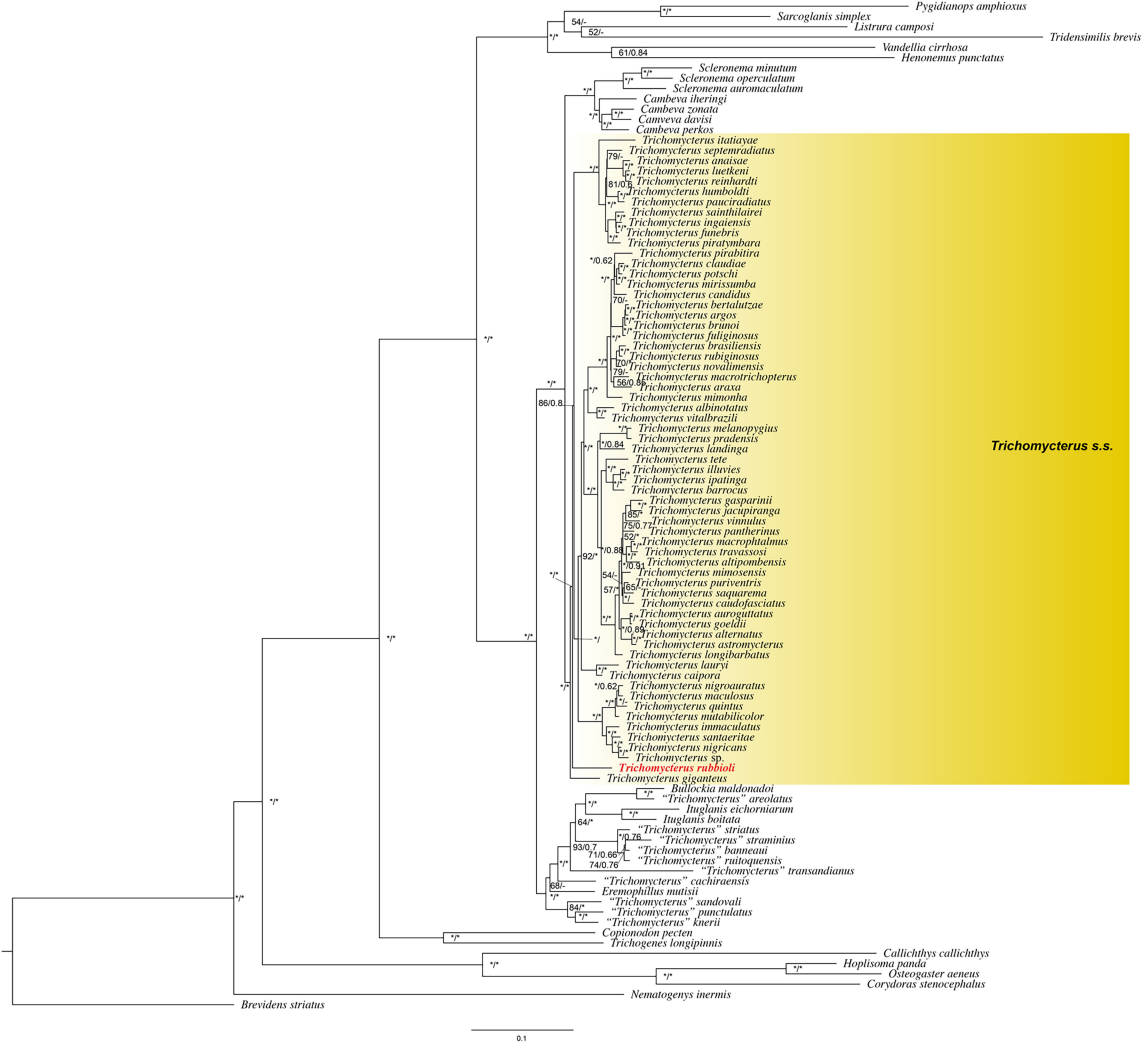
Phylogenetic relationships among 84 trichomycterine taxa and 14 outgroups, inferred by maximum likelihood (ML) from the analysis of a multigene dataset (3030 bp). Numbers on each node correspond to Ultrafast Bootstrap percentages from ML followed by posterior probability from Bayesian inference; asterisks indicate a support value above 95%, hyphens indicate support value under 50%.

**FIGURE 2:**
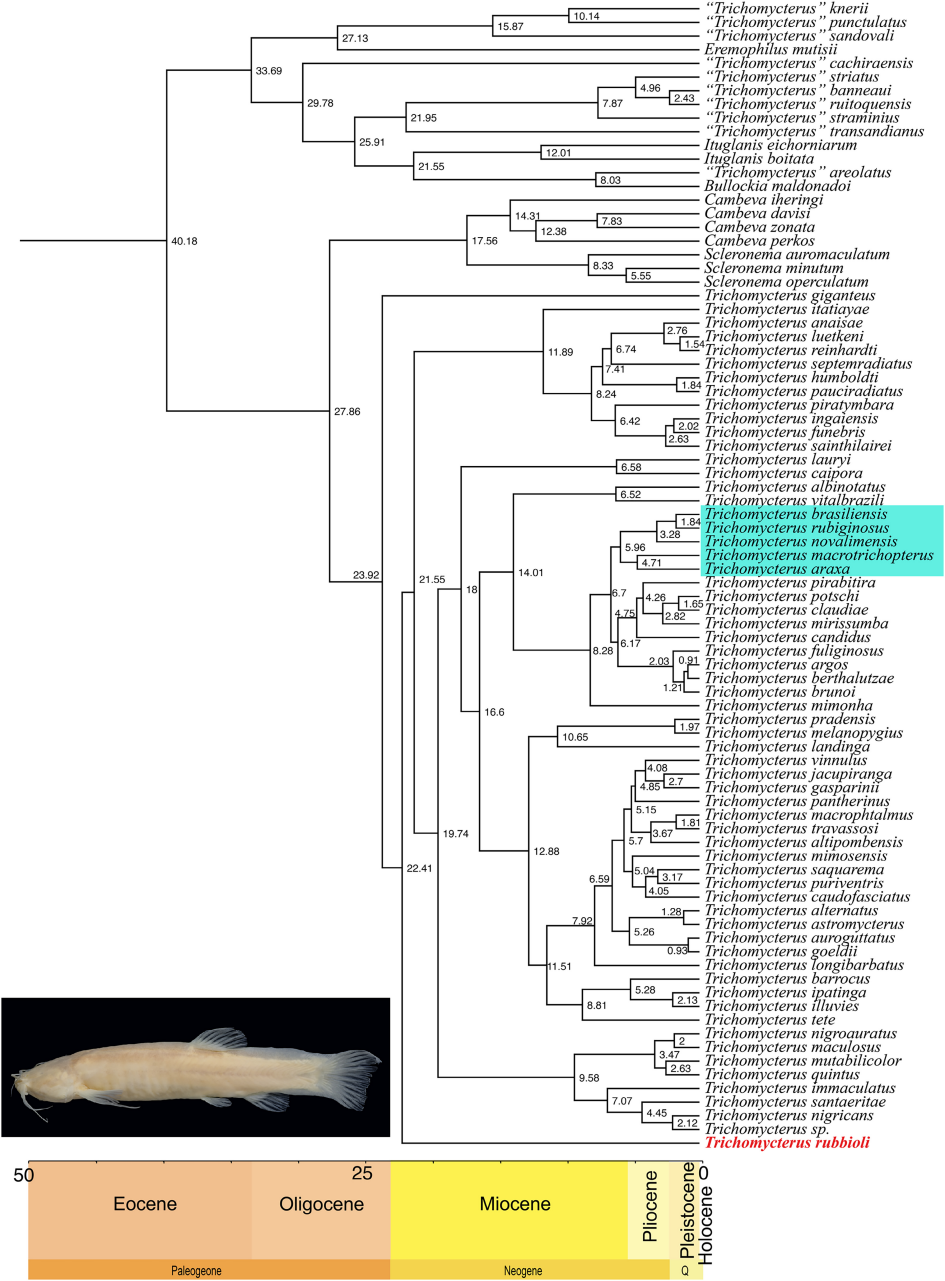
Time‐scaled tree obtained from the Bayesian analysis in Beast. Numbers near nodes represent median clade ages. Green shading indicates a *Cryptocambeva* clade with species endemic to the Rio São Francisco basin.

*Trichomycterus rubbioli* was supported as a member of the *Trichomycterus s.s*. clade as sister to a group comprising all species of *Trichomycterus s.s*. except *Trichomycterus giganteus* Lima & Costa, 2024 (Figure 1). The time‐calibrated analysis indicated a divergence time between the *T. rubbioli* lineage and its sister group in the early Miocene, approximately 22 Ma (16.3–29.0 Ma) (Figure 2).

## 4 DISCUSSION

### 4.1 Biogeographical implications

The phylogenetic analysis supported *T. rubbioli* as belonging to *Trichomycterus s.s*. (Figure 1), a clade endemic to eastern Brazil (e.g. Katz et al., 2018). Congeners and other members of the *Trichomycterus* lineage, a clade with broad geographical distribution in South America (Ochoa et al., 2020), typically live in fast‐flowing streams, in relatively cold waters of mountainous regions (Costa, 2021). The origin of the Trichomycterinae has been estimated to have occurred in the Eocene, with its lineages geographically spreading rapidly across South America from the central and northern Andes (Costa et al., 2025; the present paper), which can be explained by the Eocene global cooling (Mudelsee et al., 2014).

The origin of *Trichomycterus s.s*. has been estimated to have occurred still in the Oligocene (Vilardo et al., 2023; present paper), with biogeographic inferences indicating that this genus could already occur in a vast region of eastern Brazil before the Oligocene–Miocene boundary (Vilardo et al., 2023). Our present analysis indicated *T. rubbioli* as the only member of its lineage, which had its estimated origin in the early Miocene. The origin of this lineage is thus supported as being before the climatic period that led to the formation of the semi‐arid conditions of the Caatinga in the middle Miocene (Harris & Mix, 2002), when most dry‐adapted endemic plant lineages had their origin (Fernandes et al., 2022), as well as prior to the strong cooling period followed by aridification that marked the end of the Miocene (Herbert et al., 2016).

Caves are potential refugia for animals during climatic periods of extreme aridification, making possible allopatric divergence from epigean lineages (e.g. Ballarin & Li, 2018). In the semiarid region where *T. rubbioli* occurs, fast‐flowing streams typically dry completely during long periods of the year (W.J.E.M.C., pers. obs.), except for some streams that run inside caves (Vaz et al., 2025). The desertification event of the late Miocene, documented in various regions worldwide, may have played a crucial role in confining freshwater animal lineages to subterranean habitats; however, the origins of cave‐dwelling habits could be older (Wessel et al., 2007).

Recent studies have indicated the origin of different troglobitic lineages of arthropods from the Caatinga at the end of the Miocene (Bento et al., 2024). The absence of records of closely related trichomycterine epigean congeners in areas surrounding the cave system prevents accurate estimates of the timing of cave colonization and the subsequent troglobitic origin of the *T. rubbioli* lineage. The combination of available data supports an evolutionary scenario in which the *T. rubbioli* lineage adapted to the troglobitic lifestyle while neighbouring epigean lineages became extinct between the middle and late Miocene coinciding with the establishment and subsequent intensification of the semiarid conditions in the region. A similar hypothesis was described by Mao et al. (2021) to explain the origin of troglobitic *Sinocyclocheilus* (Cyprinidae, Barbinae) during a late Miocene aridification process in China, as well as a time‐calibrated phylogenetic study on southeastern Asia scorpions supported extinction of peripheral epigean populations during a late Miocene aridification process, while hypogean individuals survived within caves (Loria et al., 2022). Therefore, the model here adopted to explain *T. rubiolli* isolation in that cave system is plausible, but data on the geological formation of the system, presently unavailable, is necessary to support this hypothesis.

### 4.2 Relationships of cave‐dwelling trichomycterines from the caatinga

**FIGURE 3:**
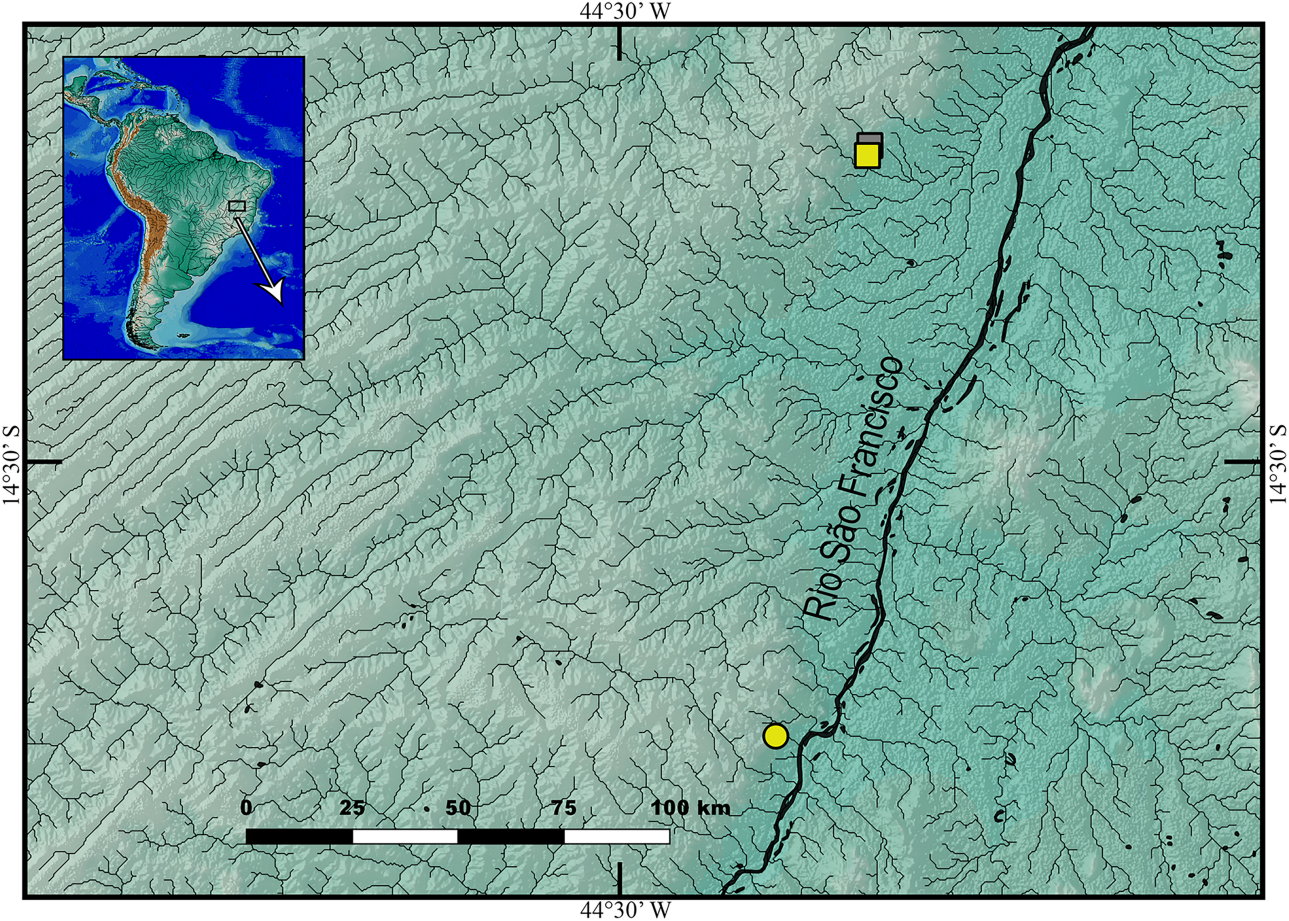
Geographical distribution of *Trichomycterus rubbioli*, squares, and *T. itacarambiensis*, circle.

In addition to *T. rubbioli*, belonging to the *Trichomycterus s.s*. clade, there is another trichomycterine, *‘Trichomycterus’ itacarambiensis* Trajano & de Pinna, 1996, with uncertain phylogenetic position but formally placed in *Trichomycterus*, that occurs about 140 km from the distribution area of *T. rubbioli* (Figure 3), in a transition area between the savanna‐like Cerrado and the semiarid Caatinga. Like *T. rubbioli*, *‘T’. itacarambiensis* inhabits streams belonging to the Rio São Francisco basin, but in this region streams around the cave system do not dry seasonally. The phylogenetic relationships of *‘T’. itacarambiensis* are still unknown, and no material was available for the present study.

The original description of *‘T’. itacarambiensis* includes only data on the external morphology (Trajano & de Pinna, 1996). Among the 25 individuals of the type series of *‘T’. itacarambiensis*, nine were true albino specimens (i.e. red‐eyed and unresponsive to 1‐DOPA) and 16 were pigmented, whereas the eye morphology varied from vestigial to fully developed eyes (Trajano & de Pinna, 1996). In an illustration of a pigmented specimen with fully developed eyes (Trajano & de Pinna, 1996: Figure 3a), it is possible to observe certain morphological character states that in combination are diagnostic for the subgenus *Cryptocambeva* Costa, 2021 of *Trichomycterus s.s*., endemic to southeastern Brazil (Costa, 2021), including a clade with species endemic to the Rio São Francisco basin (Figure 2). These character states include small eyes positioned nearer snout than to the opercular patch of odontodes, opercular patch of odontodes round and relatively small, and a colour pattern consisting of small dark grey dots that are darker along the longitudinal midline of the flank (e.g. Costa, 2021). The presence of a total of seven pectoral‐fin rays and anal‐fin origin at a vertical through the posterior third of the dorsal‐fin base are also compatible with the general morphology of species of *Cryptocambeva* and distinguishes it from most other subgenera, including basal lineages like the subgenus *Megacambeva* Costa, 2021 and *T. rubbioli*, not formally placed in any subgenus, that have nine or 10 rays (e.g. Costa, 2021). These data highly suggest that cave colonization has arisen independently in these two troglobitic trichomycterines from the Caatinga, a hypothesis that would be concordant with results obtained for hypogean trichomycterines from the northern Andes supporting multiple colonisations of the cave environment (Flórez et al., 2021).

## AUTHOR CONTRIBUTIONS

Conceptualization: W.J.E.M.C. Data obtaining and analyses: W.J.E.M.C., P.J.V. and J.L.O.M. Investigation and data curation: W.J.E.M.C., J.L.O.M., A.M.K., V.M.A.‐S., P.L.C.U. and R.L.F. Writing – original draft: W.J.E.M.C. Writing – final version: W.J.E.M.C., P.J.V., J.L.O.M., V.M.A.‐S., P.L.C.U. and R.L.F. Visualization: P.J.V. and A.M.K. Supervision: W.J.E.M.C. Funding acquisition: W.J.E.M.C. and R.L.F. All authors have read, revised and agreed to the published version of the manuscript.

## FUNDING INFORMATION

This study was supported by Conselho Nacional de Desenvolvimento Científico e Tecnológico (CNPq; grant 304,755/2020‐6 to W.J.E.M.C. and grant 302,925/2022‐8 to R.L.F.), Fundação Carlos Chagas Filho de Amparo à Pesquisa do Estado do Rio de Janeiro (FAPERJ; grant E‐26/204.305/2024 to W.J.E.M.C., 26202.327/2018 and 202.328/2018 to J.L.O.M., and E‐26/202.005/2020 to A.M.K.) and Fundação de Amparo à Pesquisa do Estado de Minas Gerais (FAPEMIG; grant 5.02/2022 to P.L.C.U.). This study was also supported by CAPES (Coordenação de Aperfeiçoamento de Pessoal de Nível Superior, Finance Code 001) through the Programa de Pós‐Graduação em: Biodiversidade e Biologia Evolutiva/UFRJ and Genética/UFRJ.

## CONFLICT OF INTEREST STATEMENT

The authors declare that they have no competing financial interests or personal relationship that could have influenced the work reported in this paper.

## Supporting information


**APPENDIX S1.** Terminal taxa and GenBank accessions numbers by gene used in molecular analyses.

**APPENDIX S2.** Best‐fitting partition schemes with the respective number of base pairs and the best‐suited evolutive models.

## Data Availability

Data sharing not applicable to this article as no datasets were generated or analysed during the current study.
