# Supplementary material for: Time‐calibrated relationships of a rare cave catfish ( Trichomycterus rubbioli ): Shedding light on troglobitic lifestyle origin in the Brazilian caatinga
Source: J Fish Biol. 2026 Mar 25;109(1):362–70. doi: 10.1111/jfb.70419 (PMC13397296; doi:10.1111/jfb.70419)
Supplement: Supplementary file 1 — APPENDIX S1. Terminal taxa and GenBank accessions numbers by gene used in molecular analyses. [file JFB-109-362-s001.docx]

**Appendix S1.** Terminal taxa, and GenBank accessions numbers by gene used in molecular analyses.

| **Species** | **Voucher ID** | **COI** | **CYTB** | **MYH6** | **RAG2** |
| --- | --- | --- | --- | --- | --- |
| *T. albinotatus* | UFRJ 9873 | MN813007 | MK123716 | MK123743 | MN812990 |
| *T. alternatus* | UFRJ 13165 | OQ357886 | OQ355710 | OQ355721 | OQ400957 |
| *T. altipombensis* | UFRJ 12770 | OP698260 | OP688466 | OP688468 | OP688472 |
| *T. anaisae* | UFRJ 9655 | MT941782 | MT941820 | — | — |
| *T. araxa* | UFRJ 12803 | — | OM250023 | — | OM250025 |
| *T. argos* | UFRJ 13146 | OQ357887 | OQ355711 | OQ355722 | OQ400958 |
| *T. astromycterus* | UFRJ 12773 | — | OK652453 | OK652451 | OK652448 |
| *T. auroguttatus* | UFRJ 10477 | MT435135 | MT436452 | MT436450 | — |
| *T. barrocus* | UFRJ 13143 | OQ357889 | OQ355713 | OQ355723 | OQ400959 |
| *T. berthalutzae* | UFRJ 13142 | OQ357888 | OQ355712 | OQ355724 | OQ400960 |
| *T. brasiliensis* | UFRJ 10642 | MK123691 | MK123717 | MK123744 | MK123763 |
| *T. brunoi* | UFRJ 10321 | MW19675 | MW196760 | MW196772 | MW196785 |
| *T. caipora* | UFRJ 14374 | — | PV929701 | PV929703 | PV929705 |
| *Trichomycterus sp.* | UFRJ 10059 | MN813008 | MN813000 | MW196760 | MN812992 |
| *T. candidus* | UFRJ 12808 | OQ357890 | OQ355714 | OQ355725 | OQ400961 |
| *T. caudofasciatus* | UFRJ 10322 | — | MK123719 | MN812995 | MK123764 |
| *T. claudiae* | UFRJ 9959 | MW196754 | MW196764 | MW196776 | MW196790 |
| *T. fuliginosus* | UFRJ 10319 | MW196750 | MW196759 | MW196771 | MW196784 |
| *T. funebris* | UFRJ 9856 | MT941785 | MT941823 | KY858121 | KY858194 |
| *T. gasparinii* | UFRJ 13140 | OQ357891 | OQ355715 | OQ355726 | OQ400962 |
| *T. giganteus* | UFRJ 11689 | MK123693 | MK123720 | MK123746 | MT446426 |
| *T. goeldii* | UFRJ 12467 | MT435136.1 | MT436453.1 | MT436451.1 | MT446427.1 |
| *T. humboldti* | UFRJ 9963 | MT941787 | MT941824 | — | — |
| *T. ingaensis* | UFRJ 9882 | MT941790 | MT941829 | — | — |
| *T. immaculatus* | UFRJ 10001 | MK123694 | MK144348 | MK123747 | MF431120 |
| *T. ipatinga* | UFRJ 13107 | OQ357892 | OQ355716 | OQ355727 | OQ400963 |
| *T. illuvies* | UFRJ 13106 | OQ357894 | OQ355717 | OQ355728 | OQ400964 |
| *T. itatiayae* | UFRJ 11926 | MW671552 | MW679291 | OL779229 | OL779233 |
| *T. jacupiranga* | UFRJ 12174 | OL764372 | OL779234 | OL779230 | OL779232 |
| *T. landinga* | LGC 1625 | — | MH621016 | — | — |
| *T. lauryi* | UFRJ 12791 | ON799208 | ON808695 | ON808696 | — |
| *T. longibarbatus* | UFRJ 13144 | OQ357895 | OQ355718 | OQ355729 | OQ400965 |
| *T. luetkeni* | UFRJ 10268 | MT941793 | MT941831 | KY858148 | KY858214 |
| *T. macrophtalmus* | UFRJ 12115 | OL741727 | OL752426 | OL752418 | OL752421 |
| *T. macrotrichopterus* | UFRJ 8320 | MW196753 | MW196762 | MW196774 | MW196787 |
| *T. maculosus* | UFRJ 12340 | MN813010 | MN813002 | MN812998 | MN812994 |
| *T. mimonha* | UFRJ 10312 | MW196749 | MW196758 | MW196770 | MW196783 |
| *T. mimosensis* | UFRJ 13139 | OQ357893 | OQ355719 | OQ355730 | OQ400966 |
| *T. mirissumba* | UFRJ 10475 | MW196752 | MW196761 | MW196773 | MW196786 |
| *T. melanopygus* | UFRJ 13164 | OQ357896 | OQ355720 | OQ355731 | OQ400967 |
| *T. mutabilicolor* | UFRJ 12641 | — | OK247576 | – | – |
| *T. nigricans* | UFRJ 10989 | MN813005 | MK123723 | MK123750 | MK123765 |
| *T. nigroauratus* | UFRJ 10305 | MK123696 | MK123724 | MK123751 | MK123766 |
| *T. novalimensis* | UFRJ 9656 | MW196755 | MW196765 | MW196777 | MW196791 |
| *T. pantherinus* | UFRJ 11015 | MK123697 | MK123725 | MK123752 | MN812989 |
| *T. pauciradiatus* | UFRJ 9875 | MT941796 | MT941833 | MW196769 | MW196782 |
| *T. pirabitira* | UFRJ 8284 | KY857982 | KY858051 | KY858133 | OL779231 |
| *T. piratymbara* | UFRJ 9884 | MT941803.1 | MT941840 | KY858121 | KY858194 |
| *T. potschi* | UFRJ 10992 | — | MW196763 | MW196775 | MW196789 |
| *T. pradensis* | UFRJ 11027 | MN813003 | MK123726 | MK123753 | MN812988 |
| *T. puriventris* | UFRJ 13141 | OP698259 | OP688465 | OP688467 | OP688471 |
| *T. quintus* | UFRJ 12022 | MT299917 | MN812999 | MT305242 | — |
| *T. reinhardti* | UFRJ 9489 | MK123698 | MK123727 | MF431106 | MF431119 |
| *T. rubiginosus* | UFRJ 9657 | MK123699 | MK123728 | MK123754 | MK123767 |
| *T. rubbioli* | UFRJ 14097 | PV899768 | PV929700 | PV929702 | PV929704 |
| *T. sainthilairei* | UFRJ 9886 | MT941814 | MT941851 | — | — |
| *T. santaeritae* | UFRJ 12408 | MN813009 | MN813001 | MN812997 | MN812993 |
| *T. saquarema* | UFRJ 12999 | OP698258 | OP688464 | — | OP688470 |
| *T. septemradiatus* | UFRJ 9888 | MK123700 | MK123729 | MK123755 | MW196781 |
| *T. tete* | UFRJ 7774 | OL741729 | MH620966 | — | — |
| *T. travassosi* | UFRJ 10314 | MK123701 | MK123730 | MK123756 | OL752425 |
| *T. vinnulus* | UFRJ 12775 | — | OK652452 | OK652450 | OK652449 |
| *T. vitalbrazili* | UFRJ 12125 | MT435137 | MK748279 | MK748280 | MT446428 |
| *Bullockia maldonadoi* | LBP3112 | KY857926 | — | KY858081 | KY858166 |
| *Cambeva davisi* | UFRJ 9762 | — | MK123714 | MK123741 | MK123762 |
| *Cambeva zonata* | LBP2653 | KY857986 | KY858053 | KY858137 | — |
| *Cambeva perkos* | LBP17033 | KY857981 | KY858050 | KY858132 | KY858202 |
| *Cambeva iheringi* | LBP4512 | KY858008 | KY858074 | KY858159 | KY858223 |
| *Eremophilus mutisii* | ANSP11306 | KY857931 | — | KY858086 | KY858171 |
| *Scleronema auromaculatum* | UFRJ 12802 | OM037445 | OM037134 | OM037135 | OM037136 |
| *Scleronema cf. operculatum* | UFRJ 11515 | MK123686 | MK123708 | MK123736 | MK123760 |
| *Scleronema cf. minutum* | UFRJ 11513 | KY857957 | KY858031 | KY858109 | KY858184 |
| *Ituglanis echorniarum* | LBP4686 | KY857939 | KY858020 | KY858094 | KY858176 |
| *Ituglanis boitata* | UFRJ 10582 | MK123684 | MK123706 | MK123734 | MK123758 |
| *Trichomycterus areaolatus* | LBP3118 | KY857964 | KY858036 | KY858115 | KY858188 |
| *Trichomycterus banneaui* | LBP19847 | KY857968 | — | KY858119 | KY858192 |
| *Trichomycterus cachiraensis* | LBP19832 | KY857971 | — | KY858122 | KY858195 |
| *Trichomycterus striatus* | LBP19846 | KY858003 | KY858069 | KY858154 | KY858219 |
| *Trichomycterus punctulatus* | ANSP180733 | KY857983 | — | KY858134 | KY858203 |
| *T. ruitoquensis* | LBP19838 | KY857984 | — | KY858135 | — |
| *T. sandovali* | LBP19833 | KY857985 | KY858052 | KY858136 | KY858205 |
| *T. straminius* | LBP19834 | KY858006 | KY858072 | KY858157 | KY858221 |
| *T. transandinus* | LBP19845 | KY858007 | KY858073 | KY858158 | KY858222 |
| *T. knerii* | LBPV-18717 | KY857987 | KY858054 | KY858138 | KY858206 |
| *Listrura camposi* | UFRJ 11400 | MK123703 | MK123732 | KY858106 | MN385824 |
| *Tridensimilis brevis* | UFRJ 11157 | — | — | MF431113 | MK123768 |
| *Pygidianops amphioxus* | UFRJ 11248 | MN385801 | — | MN385812 | MN385823 |
| *Sarcoglanis simplex* | LBP3172 | KY857956 | KY858030 | KY858108 | — |
| *Trichogenes longipinnis* | UFRJ 10295 | MK123682 | MK123704 | MF431104 | MF431117 |
| *Vandellia cirrhosa* | UFRJ11923 | MN385797 | — | MN385809 | — |
| *Henonemos punctatus* | LBP15974 | KY857933 | KY858014 | KY858088 | KY858173 |
| *Copionodon pecten* | LBP17357 | KY857929 | — | KY858084 | KY858169 |
| *Nematogenys inermis* | LBP3105 | KY857952 | — | KY858107 | KY858182 |
| *Callichthys callichthys* | ROM-T10404 | KP960058 | — | — | KP960357 |
| *Corydoras stenocephalus* | ROM-T12839 | — | KP960054 | KP960365 | KP960358 |
| *Osteogaster aeneus* | ROM-T12836 | NC_063780 | KP960056 | — | KP960360 |
| *Hoplisoma panda* | ROM-T12932 | KU568797 | KP960057 | — | KP960362 |
| *Leporinus striatus* | LBP3180 | KX086982 | KX086761 | KX086887 | KX086982 |
