# Supplementary material for: Time‐calibrated relationships of a rare cave catfish ( Trichomycterus rubbioli ): Shedding light on troglobitic lifestyle origin in the Brazilian caatinga
Source: J Fish Biol. 2026 Mar 25;109(1):362–70. doi: 10.1111/jfb.70419 (PMC13397296; doi:10.1111/jfb.70419)
Supplement: Supplementary file 2 — APPENDIX S2. Best‐fitting partition schemes with the respective number of base pairs and the best‐suited evolutive models. [file JFB-109-362-s002.docx]

Appendix S2

Best-fitting partition schemes with the respective number of base pairs and the best-suited evolutive models.

| Partition | Evolutive models for ML Analysis | Evolutive models for BI and BEAST analysis |
| --- | --- | --- |
| COI 1st; CYTB 2nd; CYTB 3rd | TIM+F+I+G4 | GTR+F+I+G4 |
| COI 2nd; MYH6 1st; MYH6 2nd | TNe+I+G4 | HKY+F+I |
| COI 3rd; | TIM+F+G4 | GTR+F+G4 |
| CYTB 1st | HKY+F+I+G4 | HKY+F+I+G4 |
| MYH6 3rd; RAG2 3rd | TPM2+F+G4 | GTR+F+G4 |
| RAG2 1st; RAG2 2nd | TIM2e+G4 | K2P+G4 |
